# Integrative Study of Genotypic and Phenotypic Diversity in the Eurasian Orchid Genus *Neotinea*

**DOI:** 10.3389/fpls.2021.734240

**Published:** 2021-10-13

**Authors:** Pavel Trávníček, Zuzana Chumová, Eliška Záveská, Johana Hanzlíčková, Lucie Kupková (Jankolová), Jaromír Kučera, Eliška Gbúrová Štubňová, Ludmila Rejlová, Terezie Mandáková, Jan Ponert

**Affiliations:** ^1^Institute of Botany, Czech Academy of Sciences, Průhonice, Czechia; ^2^Department of Botany, Faculty of Science, Charles University, Prague, Czechia; ^3^Institute of Botany, Plant Science and Biodiversity Center, Slovak Academy of Sciences, Bratislava, Slovakia; ^4^Slovak National Museum, Bratislava, Slovakia; ^5^Central European Institute of Technology (CEITEC), Masaryk University, Brno, Czechia; ^6^Department of Experimental Biology, Faculty of Science, Masaryk University, Brno, Czechia; ^7^Department of Experimental Plant Biology, Faculty of Science, Charles University, Prague, Czechia; ^8^Prague Botanical Garden, Prague, Czechia

**Keywords:** cryptic diversity, genome size, geometric morphometric, multivariate morphometric, orchids (Orchidaceae), partial endoreplication, RADseq

## Abstract

Knowledge of population variation across species’ ranges is a prerequisite for correctly assessing the overall variability of any group of organisms and provides an invaluable basis for unraveling evolutionary history, optimizing taxonomy and devising effective conservation strategies. Here, we examine the genus *Neotinea*, which represents a relatively recently delimited monophyletic genus of orchids, for which a detailed study of its overall variability was lacking. We applied a suite of biosystematic methods, consisting of flow cytometry, multivariate and geometric morphometrics, and analysis of genomic SNP data, to identify phylogenetic lineages within the genus, to delineate phenotypic variation relevant to these lineages, and to identify potential cryptic taxa within lineages. We found clear differentiation into four major lineages corresponding to the groups usually recognized within the genus: *Neotinea maculata* as a distinct and separate taxon, the *Neotinea lactea* group comprising two Mediterranean taxa *N. lactea* and *Neotinea conica*, the *Neotinea ustulata* group comprising two phenologically distinct varieties, and the rather complex *Neotinea tridentata* group comprising two major lineages and various minor lineages of unclear taxonomic value. *N. conica* constitutes both a monophyletic group within *N. lactea* and a distinct phenotype within the genus and merits its proposed subspecies-level recognition. By contrast, the spring and summer flowering forms of *N. ustulata* (var. *ustulata* and var. *aestivalis*) were confirmed to be distinct only morphologically, not phylogenetically. The most complex pattern emerged in the *N. tridentata* group, which splits into two main clades, one containing lineages from the Balkans and eastern Mediterranean and the other consisting of plants from Central Europe and the central Mediterranean. These individual lineages differ in genome size and show moderate degrees of morphological divergence. The tetraploid *Neotinea commutata* is closely related to the *N. tridentata* group, but our evidence points to an auto- rather than an allopolyploid origin. Our broad methodological approach proved effective in recognizing cryptic lineages among the orchids, and we propose the joint analysis of flow cytometric data on genome size and endopolyploidy as a useful and beneficial marker for delineating orchid species with partial endoreplication.

## Introduction

Understanding overall variability across a species’ range is a prerequisite for assessing its local variation in the appropriate context. Although the European flora is one of the best studied on Earth, comprehensive studies of individual taxa in Europe continue to provide new insights into its species diversity and discover new taxa in different plant groups (e.g., trees – Vít et al., 2017; herbs – Brock et al., 2019; Lepší et al., 2019). Orchids, on the other hand, are a special group of plants because they are a subject of detailed interest from professional and amateur botanists, and discoveries of new taxa in Europe are usually limited to the naming of local populations of dubious taxonomic value (e.g., Doro, 2020; Hirth and Paulus, 2020; Tyteca et al., 2020). Yet, the use of state-of-the-art methods in biosystematic studies of orchids has provided the opportunity to investigate complex patterns and to answer open questions about the triggers of species diversity (e.g., Bateman et al., 2018; Sramkó et al., 2019; Brandrud et al., 2020). From a biosystematic point of view, the unique process of partial endoreplication in orchids and its possible implications for taxon delimitation deserve greater attention. Partial endoreplication is a type of endopolyploidy in which only a species-specific part of the genome is replicated and individual cells in the tissue possess a specific amount of DNA reflecting the number of rounds of endopolyploidy (Leitch and Dodsworth, 2017; Brown et al., 2017). This phenomenon is known from all but one orchid subfamily (Trávníček et al., 2015) and is thought to be related to diversity in genome size and GC content (Trávníček et al., 2019). Only recently, it has been hypothesized that partial endoreplication is responsible for preferential replication of non-repetitive DNA, possibly circumventing genome size constraints (Chumová et al., 2021). Partial endoreplication patterns have also been used to delimit taxa in some orchid genera (e.g., Trávníček et al., 2011), but their implications for other orchid groups have not been tested. Moreover, some orchid genera have not yet been studied in detail, and new methodological approaches, including the evaluation of endoreplication patterns, present a tempting opportunity to study them comprehensively for the first time. An example of such an orchid group is the genus *Neotinea* Rchb.f.

This genus had originally been created to accommodate a single morphologically unique species, *N. maculata* (Desf.) Stearn and remained monotypic for more than one hundred years. However, the study of karyological features revealed that *N. maculata* exhibits striking similarities with some species formerly classified within *Orchis* s.l. (Cauwet-Marc and Balayser, 1984). DNA-based phylogenetic studies later confirmed the relationship and revealed that several taxa previously classified within the genus *Orchis* L. form a separate evolutionary lineage together with *N. maculata*, which resulted in a recircumscription of the genus (Bateman et al., 1997; Pridgeon et al., 1997; Aceto et al., 1999; Cozzolino et al., 2001; Bateman et al., 2003; Bernardos et al., 2006). However, intrageneric relationships are debated (e.g., Hürkan and Taşkın, 2021) and the use of different taxonomic concepts, usually based on knowledge of only limited variability, makes it difficult to define individual taxonomic units (e.g., species). The only well-defined species is the morphologically distinct *N. maculata*, which has also been classified as a separate section *Neotinea*. All other taxa have been classified as *Neotinea* sect. *Galericulatae* (E.Klein) F.M.Vázquez (syn. *Neotinea* sect. *Tridentatae* H.Kretzschmar, Eccarius & H.Dietr.) and can be divided into three main groups, each of them harboring entities of unclear taxonomic value.

(i) *Neotinea ustulata* (L.) R.M.Bateman, Pridgeon & M.W.Chase is frequently divided into two subspecies or varieties differing mainly in flowering time and plant height: the early flowering and usually shorter *N. u.* var. *ustulata*, and the later flowering, taller *N. u.* var. *aestivalis* (Kümpel) Tali, M.F.Fay & R.M.Bateman (e.g., Amardeilh and Dusak, 2005; Delforge, 2016; Průša, 2019; Molnár and Csábi, 2021). Although the two types may differ significantly locally, their circumscription across the distribution scale is yet to be satisfactorily resolved (Haraštová-Sobotková et al., 2005; Tali et al., 2006).

(ii) *Neotinea lactea* (Poir.) R.M.Bateman, Pridgeon & M.W.Chase occurs across the central and eastern Mediterranean, and several similar taxa have been proposed locally. *N. conica* (Willd.) R.M.Bateman exhibits only subtle morphological differences and replaces *N. lactea* in the western Mediterranean. The Sardo-Corsican endemic *Neotinea corsica* (Viv.) W.Foelsche is morphologically somewhat intermediate between *N. lactea* and *N. conica* (Foelsche and Foelsche, 2002; Amardeilh and Dusak, 2005). Another two local morphotypes have recently been distinguished in Greece (Alibertis, 2012, 2015). Currently, only *N. lactea* and *N. conica* are usually accepted at the species level (e.g., Delforge, 2016; World Checklist of Selected Plant Families [WCSP], 2021), nevertheless *N. conica* is sometimes treated as a subspecies of *N. tridentata* (e.g., Kretzschmar et al., 2007; Kühn et al., 2019).

(iii) *Neotinea tridentata* (Scop.) R.M.Bateman, Pridgeon & M.W.Chase is a widespread taxon occurring across Europe and the Mediterranean. Plants from Crete are sometimes classified as a distinct taxon named *N. tridentata* subsp. *angelica* A.Alibertis (e.g., Alibertis, 2012, 2015). Plants from Lebanon with unusually spread tepals (not forming a hood) have been described as *N. tridentata* var. *libanotica* K. Addam & M. Bou-Hamdan (Addam et al., 2014, 2016). Morphologically close is also *N. commutata* (Tod.) R.M.Bateman, which has been supposed to be a result of local hybridization (allopolyploidization) between *N. tridentata* and *N. lactea* (Pavarese et al., 2013). This taxon has been described as a Sicilian endemic, but morphologically similar individuals of unclear origin have been reported from many other areas, especially in the eastern part of the distribution range of *N. tridentata* (e.g., Petrou et al., 2011; Delforge, 2016; Tsiftsis and Antonopoulos, 2017; Angelli and Anghelescu, 2020). Currently, only two species are usually accepted: *N. tridentata* and *N. commutata* (e.g., Delforge, 2016; World Checklist of Selected Plant Families [WCSP], 2021), but sometimes the second one is synonymized with the first one (e.g., Kretzschmar et al., 2007; Kühn et al., 2019).

In our study, we explore the utility of flow cytometrically inferred traits, morphological data and restriction site-associated DNA sequences (RADseq; Baird et al., 2008) for the delimitation of taxa within the orchid genus *Neotinea*. Our main goal was to assess the suitability of common, population-targeted, biosystematic approaches for elucidating intra-generic variability with an emphasis on relationships between taxa and a re-evaluation of recent taxonomic concepts comprising four to six accepted species (e.g., Delforge, 2016; Kühn et al., 2019; World Checklist of Selected Plant Families [WCSP], 2021).

## Materials and Methods

### Plant Material

**FIGURE 1:**
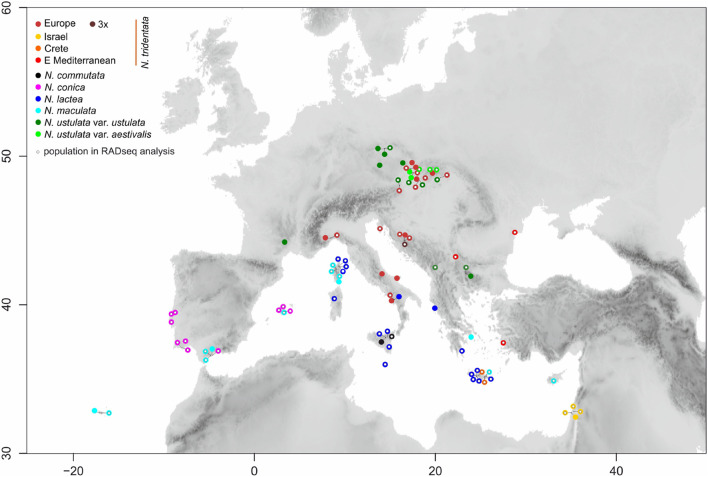
Map showing the locations of the ninety populations of *Neotinea* species included in the study. The color of filled circles corresponds to recognizable taxa/morphotypes. Smaller open circles within color ones indicate populations used in RADseq analysis.

**FIGURE 2:**
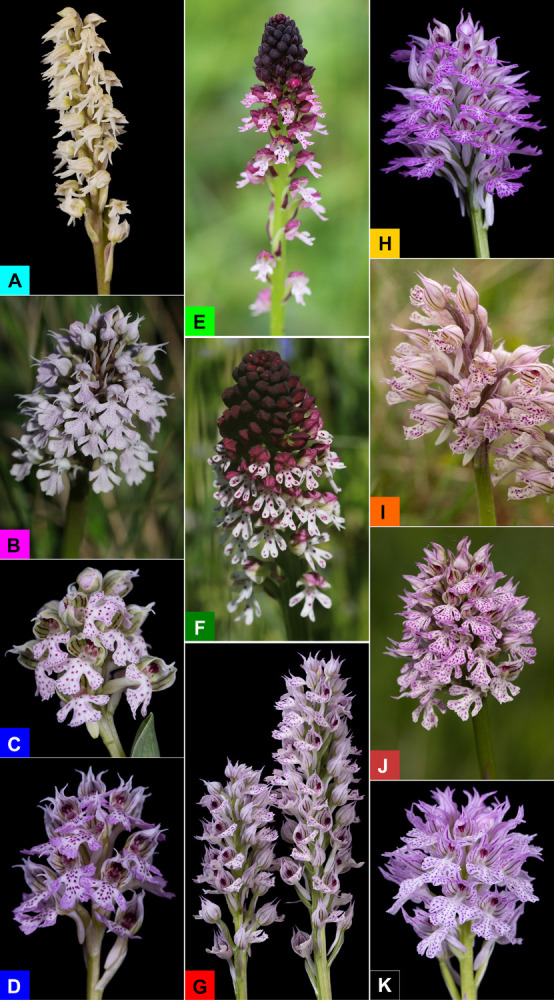
Inflorescences of ten molecularly circumscribed lineages/taxa of *Neotinea* discussed in the present phylogenetic study: *Neotinea maculata* (Cyprus, **A**), *N. conica* (Portugal, **B**), *N. lactea* (Italy, Sardinia, **C**), *N. lactea* putative hybrid (Greece, Crete, **D**), *N. ustulata* subsp. *aestivalis* (Czechia, **E**), *N. ustulata* subsp. *ustulata* (Czechia, **F**), *N. tridentata* (Romania, **G**), *N. tridentata* (Israel, **H**), *N. tridentata* (Greece, Crete, **I**), *N. tridentata* (Czechia, **J**), *N. commutata* (Italy, Sicily, **K**). The background color of the letters indicates the color used for the individual lineages in all figures throughout the manuscript.

Plant material was collected from cultivated plants of known wild origin and from native populations of *Neotinea*, in total from ninety one populations across the Eurasian area of the genus (Figures 1, 2 and Supplementary Table 1). All previous molecular-based studies supported the monophyly of the recently circumscribed genus *Neotinea* and the basal position of *N. maculata* (e.g., Hürkan and Taşkın, 2021), which may serve as an inner outgroup and objective root point. Therefore, we did not sample other outgroups, because the main objective was to explore intra-generic variation. A mini-invasive approach of collection at the individual level was applied, namely the sampling of one leaf into silica-gel for subsequent RADseq analysis and three fully developed flowers for flow cytometry analysis of the ovary(ies) and micro-morphometry of flower parts. Macro-morphometry traits were recorded *in situ* for particular individuals. Altogether 349 individuals were included in the morphometric and flow cytometry survey. For the RADseq analysis, a subset of 89 accessions from 69 populations was selected with the intention of evenly covering the geographic ranges of abundant species and to include all populations of rare taxa or morphotypes (Figure 1).

### Genome Size Estimation and Analysis of Partial Endoreplication

**FIGURE 3:**
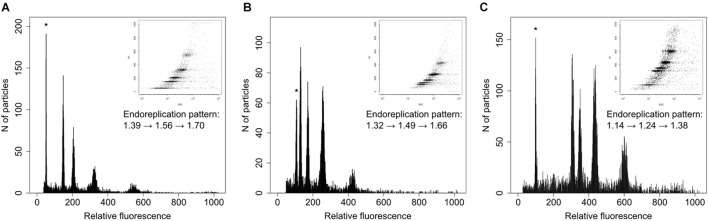
Example histograms of three taxa of *Neotinea* displaying different genome size and pattern of endoreplication. **(A)** *N. maculata* from Cyprus, **(B)** *N. ustulata* var. *ustulata* from Slovakia, and **(C)** *N. lactea* from Crete. The peaks of the internal standards (*Solanum pseudocapsicum* – **A**, *Pisum sativum* – **B,C**) are labeled by asterisks. Embedded images correspond to 2D plots of side scatter and fluorescence intensity of nuclei stained with propidium iodide. The pattern of endoreplication is given by a formula showing a genome size increase of four subsequent peaks of *Neotinea* samples.

We followed the recommendation given by Trávníček et al. (2015) and Sliwinska et al. (2021) for correct genome size estimation in orchids and preferentially analyzed ovaries and only rarely other parts (petals or young leaves). Samples were prepared by a modified two-step procedure of Otto according to Doležel et al. (2007). Briefly, a single ovary was chopped with a razor blade together with a small piece of the standard (*Pisum sativum* var. ‘Ctirad’, 2C = 8.76 pg; Trávníček et al., 2015) in a plastic Petri dish containing 0.5 ml of ice-cold Otto I buffer. The crude suspension was filtered through a nylon mesh (loop size 42 μm) into suitable cuvettes for use in flow cytometry and incubated for 5 min at room temperature. Subsequently, 1 ml of Otto II buffer supplemented with propidium iodide, RNAse (both at the final concentration of 50 mg⋅ml^–1^) and β-mercaptoethanol (4 μg⋅ml^–1^) was added, and the sample was incubated for another 5 min at room temperature before analysis. The analyses were performed using a Partec SL flow cytometer equipped with a diode-pumped solid-state 532-nm laser (Cobolt, Samba), and at least 5,000 events were recorded. The 1D histograms and 2D plots (fluorescence vs. side-scatter) were analyzed in Partec Flomax software (v. 2.4). Because of partial endoreplication in orchid tissue, all detected peaks were described in tab-delimited form to determine both ratio between the peak of the standard and the 2C peak of the sample as well as between the first (2C) and second (2C + P) peak of *Neotinea*. This allowed the estimation of the overall size and the proportion between the replicated and the non-replicated fraction of the *Neotinea*’s genomes. For the sake of simplicity, the endoreplicated part of the genome is hereafter labeled with the letter *P* and the taxon-based mean of *P* as meanP. Exemplar histograms are provided in Figure 3. Because the genome size of *N. maculata* and the primary standard (*P. sativum*) is almost identical, a secondary standard, *Solanum pseudocapsicum* (2C = 2.57 pg, estimated by repeated measurement against the primary standard), was used for all analyses of this species.

### Chromosome Counts

Young flower buds or actively growing young roots were collected from cultivated plants. The roots were pre-treated with ice-cold water for 12 h, fixed in freshly prepared fixative (ethanol:acetic acid, 3:1) for 24 h at 4 °C, and stored at −20 °C until further use. Flower buds were fixed directly in freshly prepared fixative overnight, transferred into 70% ethanol, and stored at −20 °C until use. Chromosome spreads were prepared according to the published protocol of Mandáková and Lysak (2016).

### Multivariate Morphometrics

Thirteen characters were measured *in situ* using a ruler or pair of calipers and additional 17 characters on preserved flowers in 70% ethanol under a binocular magnifier using a micro-ruler (Supplementary Table 2 and Supplementary Figure 1). Missing data were supplemented with the population mean for the missing trait. Further, 54 ratios were calculated based on primary characters (Supplementary Table 2). All measurements were selected for the best possible delineation of diagnostic characters of all taxa based on available literature. To avoid distortion of multivariate analyses, Pearson correlation coefficients were computed to reveal correlation structures and to exclude highly correlated (>0.95) characters. In fact, six primary characters and five ratios were excluded, leaving 24 primary characters and 49 ratios. Principal component analysis (PCA) based on a correlation matrix of characters and individual plants as operational taxonomic units was used to reduce the multidimensional nature of the character space and to reveal the overall pattern of morphological variation in the dataset. Canonical discriminant analysis (CDA) with individual plants assigned to groups based on multifactorial assessment using genome size, molecular analyses, geography and flowering time (to discriminate both varieties of *N. ustulata*) was performed.

### Geometric Morphometrics

From each flower preserved in ethanol, the lip (labellum), except for the spur, was removed and evenly laid out on a glass slide, dripped with water and covered with a cover-slip. Only lips from intact flowers were assessed. The prepared samples were subsequently digitized using an EOS 200D single-lens reflex camera with a TAMRON VC Di II (18–200 mm, F/3.5–6.3) lens and an external scale for calibration. First, the images were centered and consistently aligned using the program tpsDig2 v.2.31 (Rohlf, 2004). Twelve main landmarks were placed on each margin of the lip to characterize the base shape (Supplementary Figure 1). The obtained coordinates were analyzed by generalized Procrustes analysis (GPA) to normalize the shapes to an equal scale and then subjected to PCA to visualize the overall pattern of morphological lip variation according to the Klein and Svoboda protocol (Klein and Svoboda, 2017) in the R statistical environment (R Core Team, 2020) using various packages (*shapes*, *ggplot2*, *devtools*, *ellipse* and *roxygen2*; Wickham, 2016; Dryden, 2018; Murdoch and Chow, 2018; Wickham et al., 2018a, b). Finally, the photographs were processed with the image analysis software Ilastik version 1.0 (Berg et al., 2019) to capture the outline of the lip, which was subsequently evaluated by elliptic Fourier analysis of outlines (EFA). The morphometrics and statistical analyses of the overall shape were based on the modified supporting codes by Telesca et al. (2017, 2018) in the R statistical language (R Core Team, 2020) using primarily the library *Momocs* (Bonhomme et al., 2014). The outlines were extracted from gray-scale images, converted into a list of 2D pixel coordinates and normalized via outline smoothing (smoothing value used = 2), centroid alignment, point sampling along each outline (700 points for each lip), Procrustes superposition and starting point normalization. On the calibrated dataset, EFA was computed with nine harmonics comprising (99% of total harmonics power. A principal component analysis was performed to observe shape variation among species.

### DNA Extraction

Total genomic DNA was extracted from 0.5 g of silica-dried leaf material by the Sorbitol method (Štorchová et al., 2000) with two modifications: 1,600 μl of extraction buffer per sample were used (instead of 1,300 μl) and 6 μl of RNase were used instead of 4 μl in the first step. The samples were cleaned with Agencourt AMPure XP beads (Beckman Coulter Genomics, Danvers, MA, United States, ratio 1 : 0.4 DNA : beads) and the quality of the DNA was checked on 1% agarose gel.

### Generation of Restriction Site-Associated DNA Sequences Data

Altogether 69 populations, and initially 89 individuals (Figure 1 and Supplementary Table 1), were used for library preparation. Genomic DNA from each sample was quantified using a Qubit 2.0 fluorometer (Invitrogen, Carlsbad, CA, United States) and diluted to the same concentration for all individuals. An initial amount of 400 ng DNA was used in a total volume of 44.4 μl. Construction of a ddRADseq library (following Peterson et al., 2012 with some modifications of Arnold et al., 2015) consists of the following steps: digesting genomic DNA with a restriction enzyme, ligating two different barcoded adaptors on to the ends of digested fragments, size-selecting from the ligation products, and PCR amplifying the remaining subset of fragments. In the first step, only one methylation-insensitive restriction enzyme (*Hpy*CH4V) was used to generate blunt-end DNA fragments following Arnold et al. (2015). Further purification with Agencourt AMPure XP beads (ratio 1 : 1.5 DNA : beads) and quantification by a Qubit device were carried out and samples were diluted to a concentration of 100 ng⋅ml^–1^. A-tailing with Klenow Fragment (3′- > 5′ exo-) was performed according to the New England Biolabs protocol^1^, but in a total volume of 30 μl. For ligation, the adaptors that simultaneously incorporate a combinatorial in-line barcode (per Craig et al., 2008) and a standard Illumina multiplexing read index were used (Peterson et al., 2012). After ligation of the adaptors, the samples (with different adapter barcodes) were pooled, cleaned with Agencourt AMPureXP beat again (ratio 1 : 1.2 DNA : beads, two times) and size-selected fragments by Pippin Prep (Sage Science, Beverly, MA, United States). We used LabChip^®^ GX Touch 24 (PerkinElmer, Hopkinton, MA, United States) for the estimation of the size-selection range (300–370 bp finally) because of the much larger genome size of *Neotinea* compared with *Arabidopsis* (where the *Hpy*CH4V restriction enzyme was used before, Arnold et al., 2015) and to attain the optimal amount of fragments (based on a computation *in silico*).

The size-selected fragments were amplified via multiple amplification reactions for each size-selected sample (Peterson et al., 2012), purified twice by Agencourt AMPureXP beads (ratio 1 : 1.5 DNA : beads), quantified on a Qubit fluorometer and pooled in equimolar concentrations. A 1.8% agarose quality check gel was performed and final concentrations were measured on a Bioanalyzer (Agilent, Santa Clara, CA, United States).

Two DNA libraries (38 + 51 samples) were prepared and sequenced on an Illumina HiSeq 4000 instrument at Novogene, Ltd., (Cambridge, GB) using a 300-cycle kit (v.3, Illumina, Inc., San Diego, CA, United States) to obtain 150 bp paired-end reads. Raw sequence data are available in the NCBI BioProjects repository under accession No. PRJNA736952.

### Analysis of Restriction Site-Associated DNA Sequences Data

The raw reads were quality-filtered and demultiplexed according to individual barcodes using Picard BamIndexDecoder (included in the Picard Illumina2bam package^2^) and the process_radtags.pl script implemented in Stacks (Catchen et al., 2013). Finally, only 84 out of 89 accessions were processed further because of very low yield (not fitting the artificial threshold of 0.5 million raw reads) from five accessions. Because our sampling mainly consisted of diploid samples and the origin of the single tetraploid individual included was not our main focus, we used standard data analysis for diploid species using Stacks (Catchen et al., 2013). RAD loci were assembled and single nucleotide polymorphisms (SNPs) were called *de novo*, using the denovo_map.pl pipeline (Stacks version 2.53). Settings for the denovo_map.pl script [i.e., the maximum number of differences between two stacks in a locus in each sample (-M) and the maximum number of differences between loci to be considered orthologous across multiple samples (-n)] were optimized on a pilot dataset according to Paris et al. (2017). The ‘populations’ routine implemented in the software Stacks was used to extract and export the selected loci, filtered based on the number of heterozygous genotypes per locus, the maximum observed heterozygosity parameter (–max_obs_het) being set to 0.65 to avoid combining paralogs within the same RAD locus.

The output vcf file was further processed using VCFtools (Danecek et al., 2011), where the –minDP flag was used to select only genotypes with a minimum depth of 5, the –remove-indels flag to exclude sites with indels and the –max-missing flag to exclude sites with a rate of missing data greater than 30%. Two types of vcf files were created – with all SNPs per locus and with one random SNP per locus. Both vcf files edited in this way was further converted to the required formats (phylip and fasta) by the python script vcf2pyhilip.py (Ortiz, 2019) and then analyzed by RAxML (Stamatakis, 2014) and SplitsTree4 (Huson and Bryant, 2006), respectively. To infer phylogenetic relationships we computed a maximum likelihood (ML) phylogeny using RAxML v. 8.2.8 (Stamatakis, 2014). Invariant sites were removed from the original phylip format (with all SNPs per locus) using the script ‘deleteAlignColumn.pl’^3^ and Felsenstein’s ascertainment bias correction was further used to account for missing invariant sites (Leaché et al., 2015). Optimal substitution models were selected beforehand via the smart model-selection algorithm (Lefort et al., 2017). Tree searches were done using the Kimura substitution model (option -m ASC_GTRCAT –K80 –asc-corr = felsenstein; Stamatakis, 2014). The best-scoring ML tree was bootstrapped using he frequency-based stopping criterion (Pattengale et al., 2010). To explore possible reticulation in the phylogeny, a phylogenetic network using NeighbourNet analysis was performed in SplitsTree4 (Huson and Bryant, 2006). Finally, PCoA analysis of SNP data based on Euclidean distances in R was performed using the *adeneget* package (Jombart and Ahmed, 2011) to better visualize the similarity between taxa. The later two analyses were performed on a dataset containing a single random SNP per locus.

## Results

### Genome Size, Chromosome Counts and Pattern of Endoreplication

**FIGURE 4:**
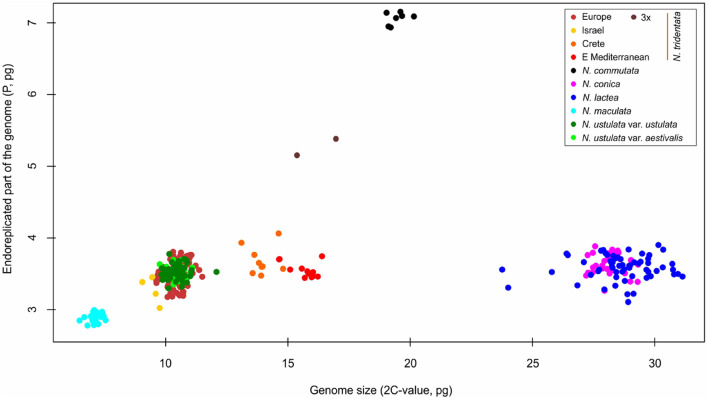
Scatter-plot of the genome size and endoreplication pattern expressed as genome size of the endoreplicated part of the genome *(P)*. Whereas all diploid taxa exhibit similar values of *P* (less than 4 pg regardless of their overall genome size), polyploids are characterized by values of *P* increased by the same fold as their ploidy level.

**FIGURE 5:**
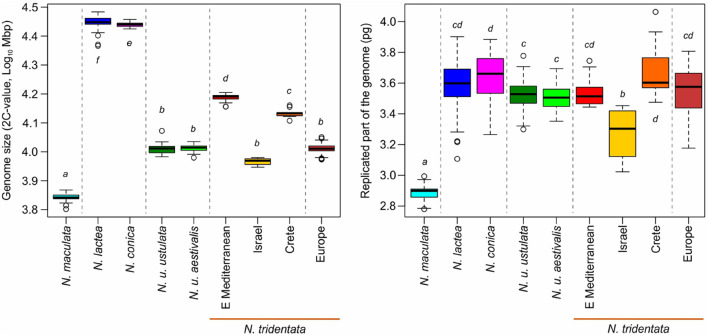
Variation in genome size **(Left)** and in the size of the replicated part of the genome **(Right)** of diploid taxa/lineages. The arrangement of taxa and their coloration follows lineages differentiated in the RAxML tree (Figure 6A), and the main clades are separated by dotted lines. The letters next to each box indicate the groups detected in Tukey’s HSD test (Table 1).

We estimated the genome sizes of all 349 individuals sampled from 91 populations. The genome size variation spanned from 6.48 pg in *N. maculata* (Tenerife, Spain) to 31.14 pg in *N. lactea* (Korfu, Greece). Despite the almost fivefold difference, both these taxa were proved to be diploids (2*n* = 42; Supplementary Figure 2). The genome size variation of all taxa and populations included in the study is presented in Table 1 and Supplementary Table 1. All of the species except *N. commutata* were previously reported to be diploid. Interestingly, despite being supposedly tetraploid (2*n* = 80, confirmed by our findings, see Supplementary Figure 2), *N. commutata* has a smaller genome than some diploids (2*C* = 19.46 pg, see Figure 4). Plotting genome size against the size of the endoreplicated part of the genome at the individual level (Figure 4) revealed unexpected geographically dependent variation in individuals assigned to *N. tridentata.* This, additionally, provided a handy tool for distinguishing between differences caused by altered ploidy level and homoploid variation (Table 1). Diploid taxa differed greatly in genome size, but the size of the endoreplicated part of their genomes was almost the same (Figure 5), the smallest mean being observed in *N. maculata* (meanP = 2.89 pg) and the greatest in *N. conica* (meanP = 3.64 pg). The change in ploidy level caused a significant shift in the size of the endoreplicated part of the genome (Table 1 and Figure 4). For DNA-triploids sampled from *N. tridentata* populations, meanP equaled 5.27 pg, which closely corresponds to a 1.5-fold increase compared with diploids. An even greater, twofold increase in meanP was observed in tetraploids of *N. commutata* (meanP = 7.06 pg).

**TABLE 1:** Variation in genome size (2*C*-value) and the size of the endoreplicated part of the genome (size corresponding to the 2*C*-value and percentage) within recognized taxa and their variants or regional evolutionary lineages revealed by phylogenetic analyses based on RADseq data (Figure 6).

Species	Ploidy	Region/var.	GS ± sd [pg]	meanP ± sd [pg]	meanP [%]	*N*
*N. commutata*	4×		19.46 ± 0.39	7.06 ± 0.09	36.3	7
*N. conica*	2×		28.14 ± 0.58^*e*^	3.64 ± 0.15^*d*^	12.9	31
*N. lactea*	2×		28.65 ± 1.43^*f*^	3.59 ± 0.17^*cd*^	12.5	60
*N. maculata*	2×		7.12 ± 0.22^*a*^	2.89 ± 0.05^*a*^	40.6	29
*N. tridentata*	2×		11.24 ± 1.82	3.55 ± 0.17	31.6	132
		Crete	13.92 ± 0.52^*c*^	3.69 ± 0.20^*d*^	26.5	9
		E Mediterranean	15.70 ± 0.54^*d*^	3.55 ± 0.10^*cd*^	22.6	13
		Israel	9.46 ± 0.30^*b*^	3.27 ± 0.19^*b*^	34.6	4
		rest of Europe	10.49 ± 0.38^*b*^	3.55 ± 0.16^*cd*^	33.8	106
	3×		16.16 ± 1.14	5.27 ± 0.17	32.6	2
*N. ustulata*	2×		10.48 ± 0.36	3.52 ± 0.09	33.6	88
		var. *ustulata*	10.46 ± 0.39^*b*^	3.53 ± 0.09^*c*^	33.7	60
		var. *aestivalis*	10.52 ± 0.29^*b*^	3.51 ± 0.09^*c*^	33.4	28

*Values for diploid lineages are accompanied by letters indicating the groups detected by multiple comparisons using Tukey’s HSD test.*

Greater variation in genome size was revealed within two of the diploid taxa, *N. lactea* and *N. tridentata* (Table 1). Whereas the example of *N. lactea* is given by a single remote measurement which likely belongs to a hybrid individual (see the results of RADseq below), the variation in *N. tridentata* is likely given by intraspecific variation where several more or less spatially isolated populations exhibit quite distinct genome sizes in comparison with the core populations in Europe (Table 1). Although precise chromosome numbers are available for a fraction of the individuals (Supplementary Figure 2), the pattern of the endoreplicated part of the genome matches well with other diploids and shows no deviation toward higher ploidy levels (Table 1 and Figure 4).

### Restriction Site-Associated DNA Sequences Assessment of Population Structure

**FIGURE 6:**
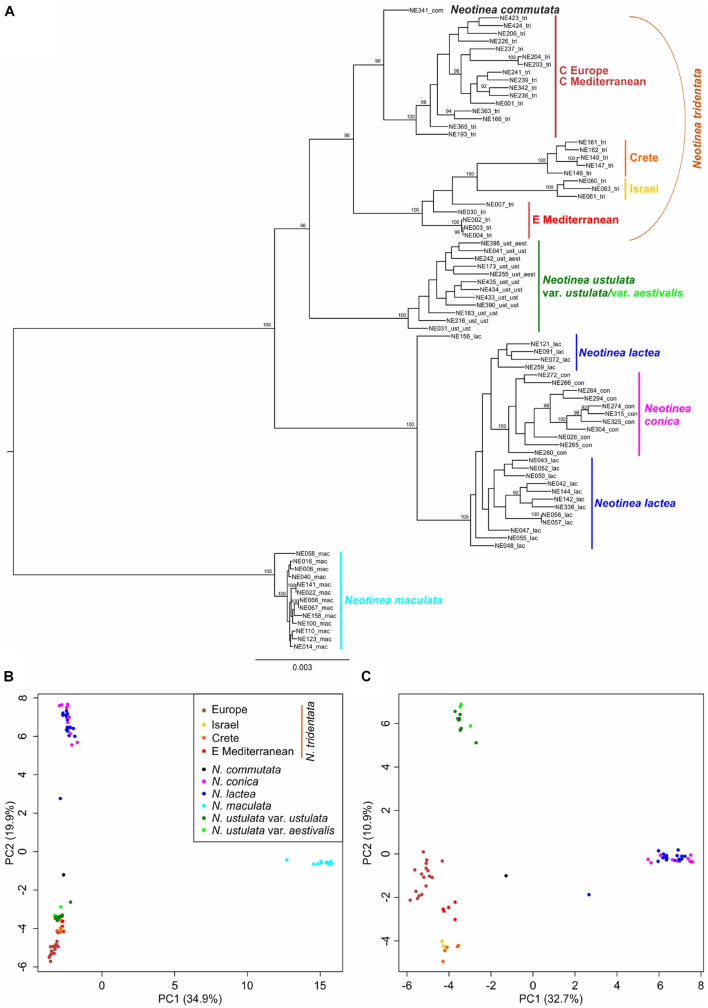
RAxML phylogeny and PCoA analyses based on RADseq-derived SNPs. **(A)** RAxML phylogeny conducted using all SNPs per locus (16,650 SNPs) is supplemented with bootstrap support (based on 450 replicates inferred from frequency-based stopping criterion; only values above 90% are shown). **(B,C)** PCoA analyses using a single random SNP per locus (3,090 SNPs) performed with *adeneget* package in R (Jombart and Ahmed, 2011) on all samples **(B)**, and without distant *N. maculata* individuals **(C)**.

After demultiplexing and filtering the raw reads, our RADseq data averaged 1.87 million (SD 0.35 million) reads for all 84 individuals involved. From a *de novo* diploid catalog built with Stacks, we retained 3,090 polymorphic loci of 141 bp. The phylogenetic analysis via RAxML was conducted on a dataset of 16,650 RADseq-derived SNPs including all samples of *Neotinea*. The PCoA ordinations and the phylogenetic network via SplitsTree were conducted on a subset of data with a single random SNP per locus and, in case of SplitsTree, without remotely placed *N. maculata*; they were analyzed to detect putative hybrids or individuals deviating in any way. The RAxML analysis (Figure 6A) showed 100% bootstrap support for the separation of *N. maculata*, which forms a sister group to the other members of the genus. Clear, fully supported, separation was also revealed for the clade including *N. lactea* and *N. conica;* however, their mutual relationship is unclear because *N. conica* represents a well-supported lineage between two sub-clades of *N. lactea* without bootstrap support. Another very well supported clade is formed by all individuals of *N. ustulata* irrespective of their variety. The most complex pattern is given by the clade, which is formed by all members of *N. tridentata* agg. together with tetraploid *N. commutata*. This clade is split into three nested clades: The first consists of mutually very well supported populations from the eastern Mediterranean, Crete and Israel, the second is formed by core populations of *N. tridentata* from Europe (Figure 1), and the third includes only one accession of *N. commutata* from Sicily.

The resulting plot given by the first two axes of the PCoA ordination of all sampled individuals based on the dataset of 3,090 SNPs (single random SNPs per locus) obtained by RADseq (Figure 6B) shows a clear separation between *N. maculata* and the rest of the genus along the first axis (explaining 34.9% of the total variation). There is also a clear separation, but along the second axis (explaining 19.9% of the total variability), between individuals of *N. lactea* and *N. conica* and individuals belonging to the group of *N. tridentata*. A better idea of the structure of these samples is provided by the ordination plot of PCoA without *N. maculata* individuals (Figure 6C), where all distinct populations within the *N. tridentata* group form more or less separate groups in the ordination space.

The neighbor network analysis in SplitsTree based on 3,090 random SNPs and a reduced dataset without remotely placed *N. maculata* revealed the same pattern (Supplementary Figure 3). Nevertheless, the analysis showed the potential hybrid origin of one individual of *N. lactea* from Crete (NE156), which is congruent with estimated genome size deviating by ∼18% from the mean of other individuals of the species used in RADseq analysis (23.8 pg vs. 28.1 pg) and also with its wider lip lobes and more horizontally oriented lips, which are characters reminiscent of Cretan plants from the *N. tridentata* group. The analysis also points to the unclear position of the only known tetraploid *N. commutata*. Its affinity to the *N. tridentata* group is obvious, but its placement close to the network basis precludes any conclusion regarding its origin.

### Multivariate Morphometrics

**FIGURE 7:**
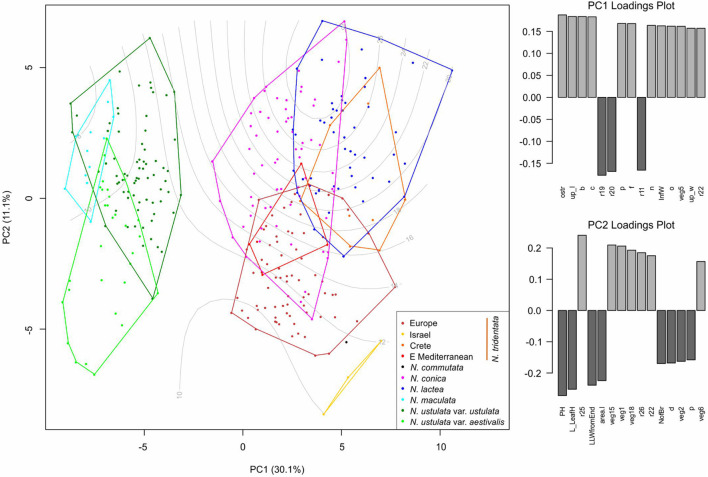
Multivariate morphometrics: principal component analysis (PCA) of 323 individuals with the full record of data. The first and second axis are displayed with the indication of explained variation. Genome size variation (values in pg DNA) is passively projected on to the ordination space using the *ordisurf* function (*vegan* R package) and shown as gray contour lines. The two right-hand panes show the ordered association of characters with the first and second PCA axis, respectively.

Full data (i.e., based on metrics of vegetative as well as floral parts) were available for 323 individuals and the final matrix consisted of 27 primary characters and 49 ratios (after removing highly correlated ones; Supplementary Table 2). The PCA analysis was performed to reveal the overall pattern between all taxa/populations that were distinguished based on independent methods (namely flow cytometry and RADseq data; Figure 7). The data show that there are two main morphological groups divided by characters tightly associated with the first PCA axis (explaining 31% of the variability), namely floral characters such as the length of the spur (ostr), mean length of the upper tepals (up_l), width of the lip (b), and width of the terminal lobe of the lip (c). Such division clearly separates *N. maculata* and both varieties of *N. ustulata* from the rest of the taxa. The second PCA axis (explaining 11.4% of the variability) is tightly associated with vegetative characters like the height of the plant (PH), length of the longest leaf (L_LeafH), length of the widest part of the leaf from its tip (LLWfromEnd), and the basal leaf area (area.l). Apparently, most taxa are quite variable in those characters and only some trends are visible, namely in the second group of taxa (involving *N. tridentata* agg., *N. lactea*, and *N. conica*). The most striking is the separation of plants from Israel, but the analysis of the only three individuals involved does not allow firm conclusions to be drawn (Figure 7).

**FIGURE 8:**
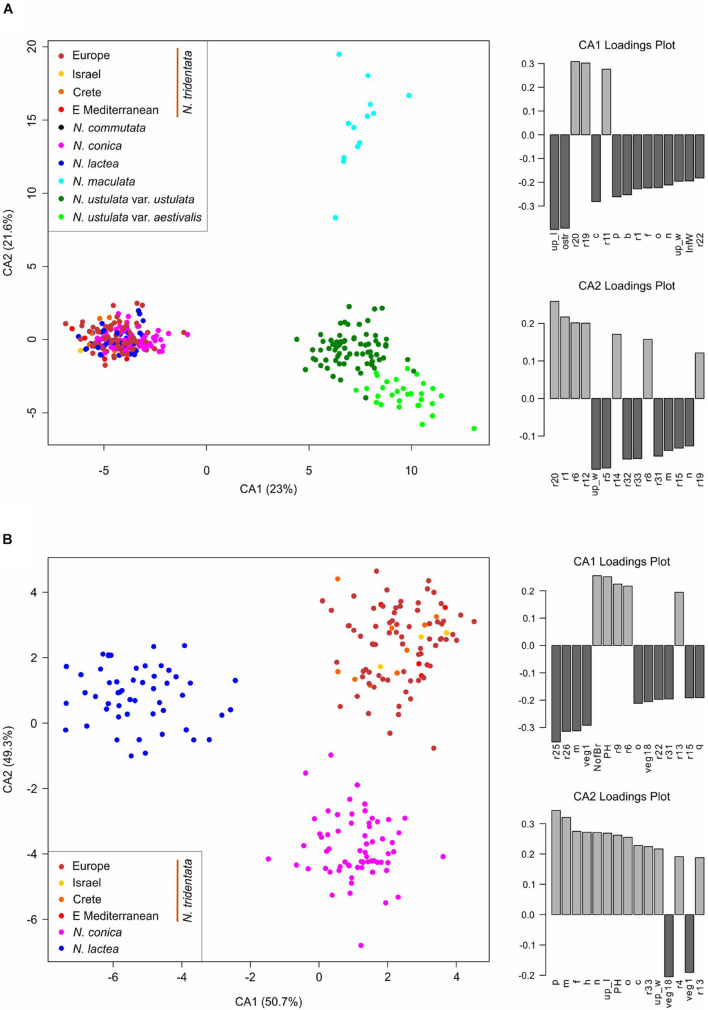
Multivariate morphometrics: canonical discriminant analysis (CDA) of **(A)** 322 individuals with the full record of data, with one accession of *N. commutata* excluded, and **(B)** 208 individuals with the full record of data, belonging to *N. conica*, *N. lactea*, and *N. tridentata*. Although the separation of populations within *N. tridentata* is retained (indicated by the different colors), the whole complex is treated as a single group (in both analyses). The two right-hand panes belonging to each ordination show the ordered association of characters with the first and second axis of the CA, respectively.

Canonical discriminant analysis (CDA) conducted on the dataset without *N. commutata* (just one individual available) and with all population of *N. tridentata* complex treated as a single group (because of the low number of individuals from the separated population of Israel, Crete and the eastern Mediterranean) revealed very good separation of *N. maculata*, both varieties of *N. ustulata* and the rest of the taxa along the first two canonical axes (Figure 8A). The most contributive characters indicating the separation of groups according to the first canonical axis (CA1) remain the mean length of upper tepals (up_l), length of the spur (ostr) and some ratios of floral characters. The second canonical axis (CA2) very well separates *N. maculata* from the rest of the species based on various ratios between floral characters (see Supplementary Table 2 for more details about the characters). We further analyzed the individuals belonging to *N. conica*, *N. lactea*, and *N. tridentata* group in a stand-alone CDA to determine the best delimiting characters (Figure 8B).

### Geometric Morphometrics

**FIGURE 9:**
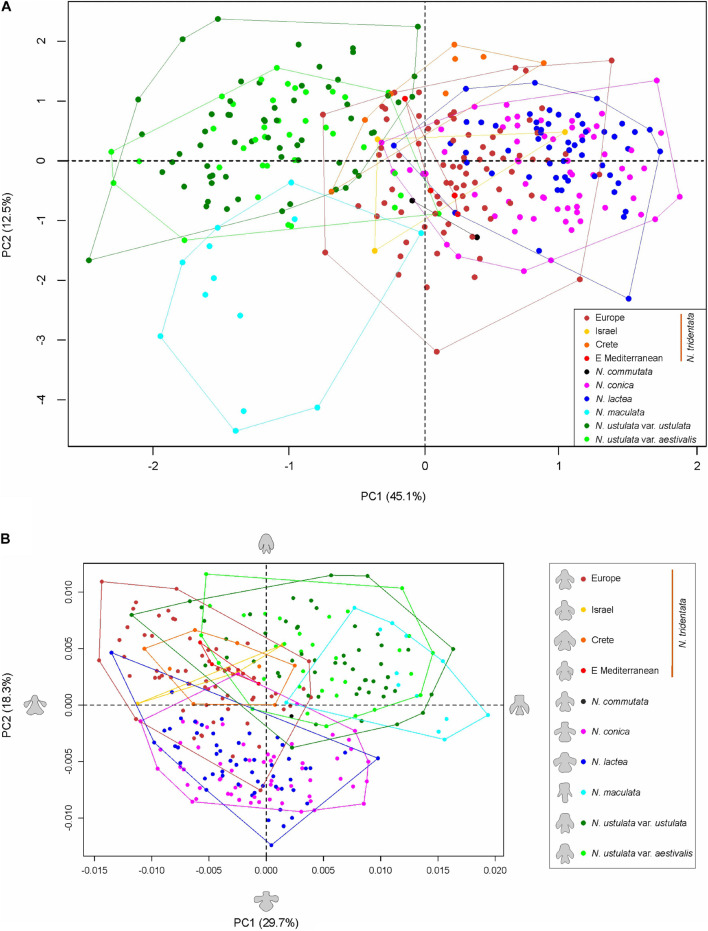
Geometric morphometrics: principal component analysis (PCA) of **(A)** normalized landmarks obtained via Generalized Procrustes analysis of 325 individuals of all *Neotinea* taxa; **(B)** lip shapes determined by Fourier analysis of outlines and usage of nine harmonics of 292 individuals of all *Neotinea* taxa. The right-hand pane shows the averaged shape of the lip of all included taxa and the color legend.

Because of the common usage of lip shape in orchid taxonomy, we also analyzed this floral part by landmark-based as well as shape-based geometric morphometrics. Generalized Procrustes analysis based on 12 landmarks revealed the trend of separation of *N. maculata* into a stand-alone group based on the first two PCA axes and of both varieties of *N. ustulata* from other taxa based on the first PCA axis (Figure 9A). A similar scenario was also revealed in the shape analysis via Fourier analysis of outlines, where the separation of the aforesaid taxa along the first PCA axis is the most apparent (Figure 9B). This analysis also provides an opportunity to estimate the averaged shape of the lip for defined groups and shows general trends of shape variability among all taxa (Figure 9B, right pane).

## Discussion

We applied a suite of biosystematic approaches consisting of a flow cytometric and karyological survey, restriction site-associated DNA sequencing, and multivariate and geometric morphometrics to understand the diversity and taxonomic complexity within *Neotinea*, a relatively recently circumscribed orchid genus (Bateman et al., 1997; et seq.). Although species belonging to this genus are well known throughout their range, a comprehensive study revealing the relationships between them based on robust sampling in this area has been lacking. This has led, among other things, to the description of many local taxa without placing them in a broader context and usually on the basis of minor differences of unclear utility for identification and of unknown variability across the range of this genus. For this reason, the four to six recently accepted species (e.g., Delforge, 2016; Kühn et al., 2019; World Checklist of Selected Plant Families [WCSP], 2021), are accompanied by dozens of ‘small’ taxa of completely unknown taxonomic value. However, this situation probably reflects the lack of a detailed study, so we have decided to provide the first comprehensive insight into the genus *Neotinea* across its range.

### What Can Be Inferred for the Genus *Neotinea*?

Our results unequivocally support the separation of four main groups within *Neotinea*: the earliest-diverged species *N. maculata*, the *N. lactea* group, *N. ustulata* and the *N. tridentata* group.

Since its establishment, *N. maculata* represents the first and most readily distinguishable species of the genus. The results of our study corroborate its separate status at the species rank. It differs from the rest of the taxa in genome size and the extent of the endoreplicated part of the genome (Figures 4, 5), occupies a distant position in our SNP-based phylogeny (Figure 6A), forms a distinct group in SNP-based ordination (Figure 6B) and has a distinct morphology, as demonstrated by all our morphometric analyses (Figures 7–9). These findings are in line with available data on seed morphology (Gamarra et al., 2007), pollination strategy, which includes nectar-rewarding flowers, albeit with a high proportion of self-pollination (van der Cingel, 1995), as well as with previous phylogenies based on candidate-gene sequencing (Pridgeon et al., 1997; Aceto et al., 1999; Bateman et al., 2003).

The *N. lactea* group includes *N. conica*, which contradicts the occasionally used taxonomic classification of this taxon under *N. tridentata* (e.g., Bateman et al., 1997; Kühn et al., 2019). However, the relationship between *N. lactea* and *N. conica* is somewhat puzzling. *N. conica* is monophyletic but nested within *N. lactea*. Four plants from Corsica form a somewhat separate lineage of *N. lactea*, sister to *N. conica*, but only weakly supported (Figure 6). The taxonomic classification of Corsican plants will require further study. A local taxon has been described as *N. corsica*, differentiated mostly by a less convex lip (up to slightly concave), being morphologically intermediate between *N. lactea* and *N. conica* (Foelsche and Foelsche, 2002). However, the extent of its morphological separation from *N. lactea* is unclear, which is mirrored in inconsistent records of its distribution. It is not known whether *N. corsica* occurs also in Sardinia and whether true *N. lactea* s.s. occurs in Corsica (e.g., Bournérias and Prat, 2005; Delforge, 2016). In our phylogenetic analysis, all plants from Sardinia grouped with *N. lactea* from other areas, which may indicate some level of evolutionary separation of Corsican plants. However, PCoA ordination of SNPs (Figures 6A,B) and morphometric data show no separation of Corsican plants from the remainder of *N. lactea*, indicating that *N. corsica* likely represents an isolated but infraspecific lineage of *N. lactea* (Supplementary Figure 6A). The phylogenetic placement of *N. conica* within *N. lactea* could be a result of a single colonization event of the westernmost part of the range that led to a novel morphotype evolved from plesiomorphic *N. lactea* (including the Corsican morphotype). Our data show clear differentiation at the morphological level, as visualized by our PCA of individuals of the *N. lactea* group (Supplementary Figure 6A) and discriminant analyses of individuals belonging to *N. tridentata* and the *N. lactea* group (Figure 8B). The most differentiating traits are found in floral parts, the most conspicuous being the shape of the lip (see also Figure 9). However, taking into account the relative subtlety of the identified morphological differences, classification at an infraspecific level seems more appropriate. Such a classification has already been proposed under the genus *Orchis* (Kreutz, 2004a; Baumann and Lorenz, 2005), but not under the recently accepted genus *Neotinea*. For this reason, we propose a new combination below. However, understanding the origin of *N. conica* and its possible migration across the Mediterranean is a subject for detailed phylogeographic research. An initial hypothesis could be long-distance dispersal from Corsica to the Balearic Islands and then to the Iberian Peninsula, following the pattern of other species (e.g., from the genus *Thymus*: Molins et al., 2011; and *Cymbalaria*: Carnicero et al., 2017).

*Neotinea ustulata* is traditionally divided into var. *ustulata* and var. *aestivalis*. Our morphometric analyses more or less confirm the clear morphological separation of these two morphotypes (Figures 7, 8A and Supplementary Figure 4B), but our phylogenetic data provide no evidence of their separation. This situation likely reflects the repeated origin of the genetically poorly differentiated var. *aestivalis* from the plesiomorphic var. *ustulata*, as hypothesized based on AFLP data by Tali et al. (2006). Conversely, convergent differentiation could likely be due to some general selection pressure or predisposition, which merits further investigation (Tali et al., 2006). Based on the data from our study, it seems that morphological differentiation is probably an adaptive response to changing conditions during the growing season (its peak flowering differs by about 6–8 weeks between the two varieties – Haraštová-Sobotková et al., 2005; Harrap and Harrap, 2010) rather than genetic differentiation. However, a detailed study focusing on a wider range of populations is needed to resolve this interesting discrepancy.

The *N. tridentata* group splits into two main well-separated lineages and *N. commutata* in a nested position (Figure 6A and Supplementary Figure 3). One lineage comprises plants from the Eastern Mediterranean (the Balkans and Turkey) and two distant well supported nested lineages corresponding to plants from Crete and from Israel, respectively. The second lineage comprises plants from Central Europe and the central Mediterranean, which seem to be related to each other. The only tetraploid species *N. commutata* exhibits a close relationship to this lineage. All these lineages are clearly separated from each other even in the ordination based on SNP data (Figures 6A,B).

Cretan populations of *N. tridentata* differ also morphologically; they are shorter, with usually smaller numbers of smaller and brightly colored flowers (Figures 7, 9A and Supplementary Figure 5). Because of these differences, they were described as *N. tridentata* subsp. *angelica* (Alibertis, 2012). However, the somewhat separate status of Cretan populations needs to be confirmed in the context of thorough sampling, namely in mainland Greece, Turkey and on other Mediterranean islands. At the level of current knowledge, Cretan populations appear to be a product of long-term isolation.

Plants from Israel markedly differ from others at first glance by unusually bright and intensive colors of their large flowers and with very early flowering in March. Similar early flowering plants occur also in other areas of the Levant around the eastern coast of the Mediterranean Sea, in Lebanon, Syria and the Hatay province of Turkey (e.g., Vela and Viglione, 2015). These populations are adjacent to other populations of *N. tridentata* in Turkey and Kurdistan regions, which flower significantly later (April – May) and which markedly differ in smaller flowers of less intensive color (e.g., Kreutz and Colak, 2009; Vela et al., 2013). The different flowering time together with separated distribution can likely act as a reproductive barrier and the Levant population could deserve taxonomic separation at some infraspecific level. The only other nearby population on Cyprus flowers also in March (and the first half of April; Kreutz, 2004b) and it could be related to the Levant population; however, it is now almost extinct and was not included in our study. From the Levant area, *N. tridentata* var. *libanotica* has been described, differentiated by unusually spread tepals not forming a hood (Addam et al., 2014, 2016). We were unable to include this material in our study, but such a position of tepals could be a local variation with little taxonomic value. Moreover, plants of var. *libanotica* grow together with classical *N. tridentata* individuals (Addam et al., 2014) and they share all other characters with the local morphotype of *N. tridentata*. Therefore, the most parsimonious solution seems to be a taxonomic broadening of var. *libanotica* to include the whole Levant population of *N. tridentata*, irrespective of tepal position. However, more plants need to be studied to avoid premature taxonomic changes.

The Eastern Mediterranean group (referred to as eastern Mediterranean in all analyses) includes plants from Serbia, Turkey and Romania and forms the basal group of the whole clade with the plants mentioned above (Figure 6A) and clearly separated group in SNP-based ordination (Figure 6C). Whereas plants from Romania seem to form a separate group, the other plants show transitions toward other nested clades (Israeli and Cretan). On the other hand, all plants from the eastern Mediterranean share the same or very close genome size that is significantly larger than in the rest of the *N. tridentata* group (Figures 4, 5 and Table 1). Their genome size is more consistent with triploid plants, but their endoreplication pattern and chromosome number point to diploids (Figure 4 and Supplementary Table 3). These plants are morphologically similar to regular *N. tridentata* of the next clade (Figures 7, 9 and Supplementary Figure 5). On the other hand, their different genome size deserves further attention and probably indicates the existence of a somewhat distinct lineage within *N. tridentata*, with an unresolved relationship to the rest of the group.

The second major clade of *N. tridentata* revealed in our study includes plants from Central Europe, Italy, Croatia and Bosnia and Herzegovina supplemented by the tetraploid *N. commutata* from Sicily (Figures 1, 6). Although the phylogenetic position and classification of *N. commutata* is somewhat unclear (Supplementary Figure 4), its chromosome number (Supplementary Figure 2) is consistent with its previously assumed tetraploid origin (Pavarese et al., 2013). On the other hand, the suggested allotetraploid origin where *N. lactea* is proposed as a possible parent is doubtful. Both the tetraploid genome size and the endoreplication pattern correspond to a two-fold value of *N. tridentata* (Figures 4, 5) and, virtually, it only naturally extends the polyploid lineage given by diploids and triploids of *N. tridentata* (Figure 4). By contrast, one can speculate that the proposed tetraploid product of hybridization between *N. tridentata* and *N. lactea* (Pavarese et al., 2013) would almost certainly have twice the genome size of *N. commutata* and a pattern of endoreplication approximately halfway between the putative parents, as is typical of hybrids (Trávníček et al., 2011; Brown et al., 2017). Our data therefore likely suggest an autotetraploid origin from *N. tridentata*. In fact, we were able to include only a single individual in our phylogenetic analyses, and better sampling is needed to address the issue once and for all.

The rest of the *N. tridentata* clade comprises plants from Europe with quite similar morphology (Figures 7, 8B, 9 and Supplementary Figure 5), without any notable structure in the phylogeny (Figure 6A and Supplementary Figure 3) and SNP-based ordination (Figures 6B,C), and with the same genome size (Figures 4, 5). These plants could very likely be classified as *N. tridentata sensu stricto.* Only in these populations relatively rare DNA-triploids were detected (Figure 4).

Finally, it must be noted that the taxonomic classification of the lineages within the *N. tridentata* group is complicated and requires a thorough study based on a broader sampling of plants from the whole distribution range.

### Taxonomic Implications of Endoreplication

Genome size alone is widely used as a species-specific marker for delineating taxa (e.g., Chumová et al., 2015; Habibi et al., 2018; Hodálová et al., 2020) and, in combination with the size of the endoreplicated part of the genome in the process of partial endoreplication, represents an unexplored possibility to delineate orchid taxa based on a single flow cytometry method. To our knowledge, this approach has been applied only rarely so far, for example, in the genus *Gymnadenia* (Trávníček et al., 2011), and its broader relevance for orchid taxonomy remains unanswered. Our study of the genus *Neotinea* thus provides another example of the useful combination of genome size with the size of the endoreplicated part of the genome for the recognition not only of specific taxa, but also for the identification of neopolyploids (Figure 4). To some extent, it also allowed us to reveal cryptic taxa or at least to point out the existence of otherwise indistinguishable variations within the *N. tridentata* group. This suspicion was finally confirmed by RAD sequencing (Figure 6). The size of the endoreplicated part of the genome appears to have broader evolutionary implications, as its uniformity among evolutionary lineages (Figure 3 and Table 1), except in recent polyploids, suggests some constraint on this trait, and the evolution of genome size is restricted to the non-endoreplicated part. This is consistent with the findings of Chumová et al. (2021), who showed different rates of evolution for the endoreplicated and non-endoreplicated parts of genome in orchids from the subtribe Pleurothallidinae.

### Restriction Site-Associated DNA Sequences – A Tool for Understanding the Evolution of Complicated Plant Groups

Much of the diversity in many orchid genera is thought to be the result of either a rapid process of radiation or complex evolution involving hybridization and polyploidization which is usually accompanied by low levels of genetic differentiation. This is commonly reflected in the poor or missing resolution of phylogenies based on DNA fragments used in botany for decades (ITS and selected cpDNA markers). This inadequacy is evident in genera where fast morphological differentiation has led to the origin of multiple species but with insufficient support in traditional phylogenies (e.g., *Ophrys* – Devey et al., 2008; Breitkopf et al., 2015; Bateman et al., 2018, 2021; *Epipactis* – Bateman et al., 2005). These days, we are witnessing an increasing application of high-throughput sequence data based phylogenies in many areas of botanical research, where the genotyping-by-sequencing (RADseq) approach has been successfully applied to tackle complex plant groups undergoing for example recent rapid radiation (e.g., Fernández-Mazuecos et al., 2018; Spriggs et al., 2019; Okuyama et al., 2020). Not surprisingly, the same way of grasping a complex history is used to advantage in orchid research (e.g., *Ophrys* – Bateman et al., 2018; *Epipactis* – Sramkó et al., 2019; *Gymnadenia* – Brandrud et al., 2019; *Dactylorhiza* – Brandrud et al., 2020; *Cycnoches* – Pérez-Escobar et al., 2020). Our study thus fits well into a newly established direction in the study of the complex evolution of some orchid groups. Moreover, our approach showed that a simplified double digest RAD method using a single blunt-end restriction enzyme, originally used for research of small genomes of *Arabidopsis* (Arnold et al., 2015 and followers), is applicable more broadly. Even though *Neotinea* genomes are up to 70-fold larger (27–30 pg for *N. lactea* vs. 0.4–0.5 pg for *Arabidopsis* diploids – Lysak et al., 2009), the use of an appropriate restriction enzyme and precise fragment size selection allowed us to successfully use such an approach in orchid research for the first time.

### A New Path in Comprehensive Orchid Research?

This study originally builds on our long-term research on orchids, specifically the causes and consequences of the unique trait of partial endoreplication (Trávníček et al., 2015, 2019; Hřibová et al., 2016; Chumová et al., 2021). By investigating the pattern of endoreplication in many orchid genera, we found that the smallest amount of the endoreplicated part of the genome in all orchids hitherto studied is observed in the *N. lactea* group (∼12–13%). In addition, other species of the genus have been found to have different but species-specific percentages of endoreplicated parts of the genome, which decrease with increasing genome size (Table 1). This finding stood at the very beginning of the research presented here, which progressively employed new methods of contemporary plant biosystematics to achieve the broadest possible view of the hidden diversity of the genus *Neotinea*. The purpose of our approach was not only to reveal the association of the pattern of endoreplication with morphological differentiation and evolution in a given orchid genus, but also to break new ground in the study of orchids with partial endoreplication in general.

## Taxonomy

*Neotinea lactea* subsp. *conica* (Willd.) J. Ponert, P. Trávníček & Chumová, *comb. nov.*

Homotypic synonyms:

Bas.: *Orchis conica* Willd., Sp. Pl., ed. 4, 4: 14 (1805).
Syn.: *Odontorchis conica* (Willd.) D.Tyteca & E.Klein, J. Eur. Orch. 40: 544 (2008).
Syn.: *Orchis lactea* var. *conica* (Willd.) H.Baumann & R.Lorenz, J. Eur. Orch. 37: 729 (2005).
Syn.: *Orchis lactea* subsp. *conica* (Willd.) Kreutz, Kompend. Eur. Orchid.: 124 (2004a).
Syn.: *Neotinea conica* (Willd.) R.M.Bateman, Bot. J. Linn. Soc. 142: 12 (2003).
Syn.: *Orchis tridentata* subsp. *conica* (Willd.) O.Bolòs & Vigo, Fl. Man. Paísos Catalans 4: 639 (2001).

Heterotypic synonyms:

Syn.: *Neotinea tridentata* subsp. *conica* (Willd.) R.M.Bateman, Pridgeon & M.W.Chase, Lindleyana 12: 122 (1997).
Syn.: *Orchis pusilla* D.Tyteca, Orchidophile (Deuil-la-Barre) 62: 628 (1984).
Syn.: *Orchis lactea* subsp. *broteroana* (Rivas Goday & Bellot) Rivas Goday, Veg. Fl. Cuenca Extrem. Guadiana: 713 (1964).
Syn.: *Orchis broteroana* Rivas Goday & Bellot, Anales Jard. Bot. Madrid 6(2): 189 (1946).
Syn.: *Neotinea conica* var. *ricardina* F.M.Vázquez, J. Eur. Orch. 40: 706 (2008).
Syn.: *Neotinea conica* var. *saenzii* F.M.Vázquez, J. Eur. Orch. 40: 707 (2008).
Syn.: *Neotinea conica* f. *gelpiana* F.M.Vázquez, Folia Bot. Extremadur. 3: 96 (2009).
Syn.: *Neotinea conica* f. *rosea* F.M.Vázquez, Folia Bot. Extremadur. 3: 96 (2009).
Syn.: *Orchis conica* f. *gelpiana* (F.M.Vázquez) Hervás, De Bellard, Calzado, J.C.Huertas, Reyes Carr. & Ruíz Cano, Micobot. Jaén 14(1): 17 (2019).
Syn.: *Orchis conica* var. *ricardina* (F.M.Vázquez) Hervás, De Bellard, Calzado, J.C.Huertas, Reyes Carr. & Ruíz Cano, Micobot. Jaén 14(1): 17 (2019).
Syn.: *Orchis conica* f. *rosea* (F.M.Vázquez) Hervás, De Bellard, Calzado, J.C.Huertas, Reyes Carr. & Ruíz Cano, Micobot. Jaén 14(1): 17 (2019).
Type:—PORTUGAL. Willdenow 16809 (B: -W 16809 -01 0!).

## Data Availability Statement

The datasets presented in this study can be found in online repositories. The names of the repository/repositories and accession number(s) can be found below: https://www.ncbi.nlm.nih.gov/, PRJNA736952.

## Author Contributions

PT and JP designed the study. PT, ZC, JH, LK, JK, EG, LR, and TM collected the data. PT, ZC, EZ, and JP analyzed the data. PT, ZC, and JP drafted the manuscript. All authors contributed to the article and approved the submitted version.

## Conflict of Interest

The authors declare that the research was conducted in the absence of any commercial or financial relationships that could be construed as a potential conflict of interest.

## Publisher’s Note

All claims expressed in this article are solely those of the authors and do not necessarily represent those of their affiliated organizations, or those of the publisher, the editors and the reviewers. Any product that may be evaluated in this article, or claim that may be made by its manufacturer, is not guaranteed or endorsed by the publisher.

## Footnotes

1
www.neb.com/M0212

2
https://github.com/wtsi-npg/illumina2bam

3
https://www.biostars.org/p/55555/

## Supplementary Material

Supplementary Figure 1 Morphological traits recorded on flowers and landmarks on the flower lip.

Supplementary Figure 2 Meiotic and mitotic chromosome spreads of selected individuals of *Neotinea.*

Supplementary Figure 3 NeighbourNet based on 3,090 random SNPs.

Supplementary Figure 4 PCA biplot of data subset including **(A)** individuals of *N. lactea* group and **(B)** *N. ustulata.*

Supplementary Figure 5 PCA biplot of data subset of all subgroups of *N. tridentata.*

Supplementary Table 1 Overview of the populations used in the study supplemented with flow cytometry results.

Supplementary Table 2 Overview of morphological characters used for multidimensional morphometry.
