# Supplementary material for: Integrative Study of Genotypic and Phenotypic Diversity in the Eurasian Orchid Genus Neotinea
Source: Front Plant Sci. 2021 Oct 13;12:734240. doi: 10.3389/fpls.2021.734240 (PMC8570840; doi:10.3389/fpls.2021.734240)
Supplement: Supplementary Figure 1 — Morphological traits recorded on flowers and landmarks on the flower lip. [file Data_Sheet_1.PDF]

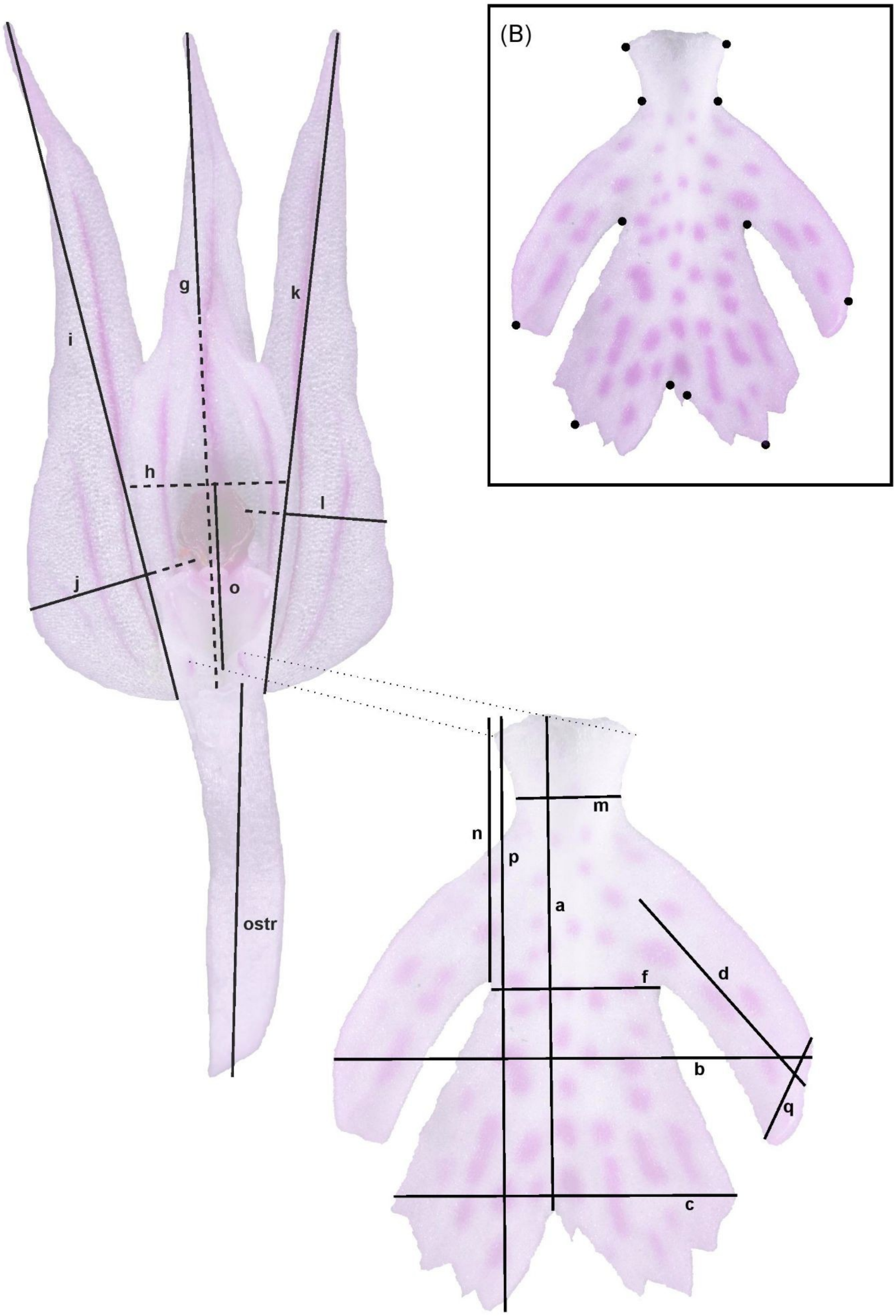

Supplementary Figure 1 | Scheme of the flower parts (outer tepals with spur and gynostemium – left; labellum / lip – bottom right) with the measurements recorded for multivariate morphometrics. **(B)** Labellum / lip supplemented with landmarks (dots) used in geometric morphometrics.

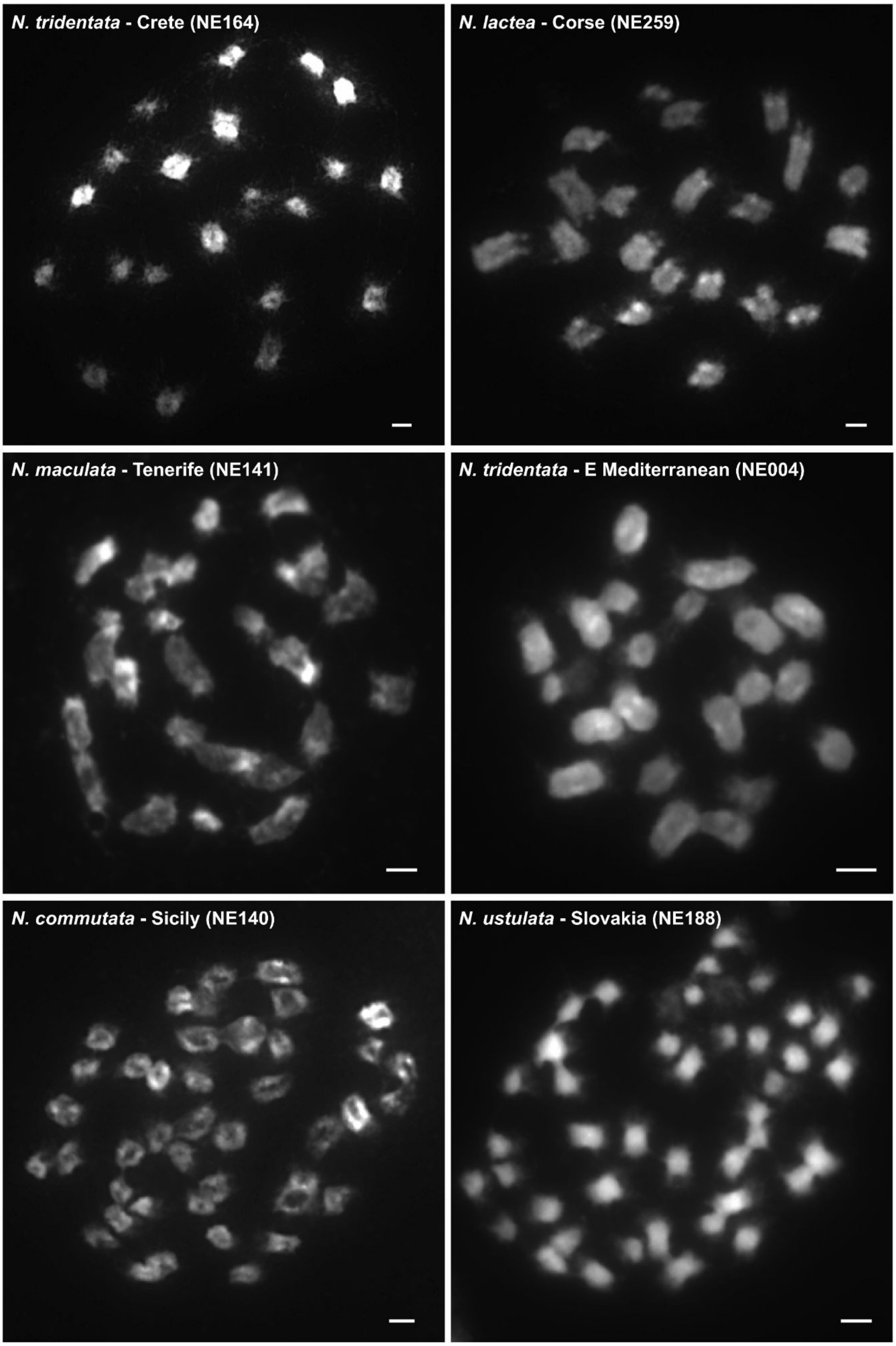

Supplementary Figure 2 | Meiotic chromosome spreads of *N. tridentata* (Cretan lineage, n = 21), *N. lactea* (n = 21), *N. maculata* (n = 21), *N. tridentata* (E Mediterranean lineage, n = 21) and *N. commutata* (n = 40) showing chromosome pairs in diakinesis obtained from flower buds and mitotic chromosome spread of *N. ustulata* (2n = 42) obtained from the root tip, scale bars = 10 µm.

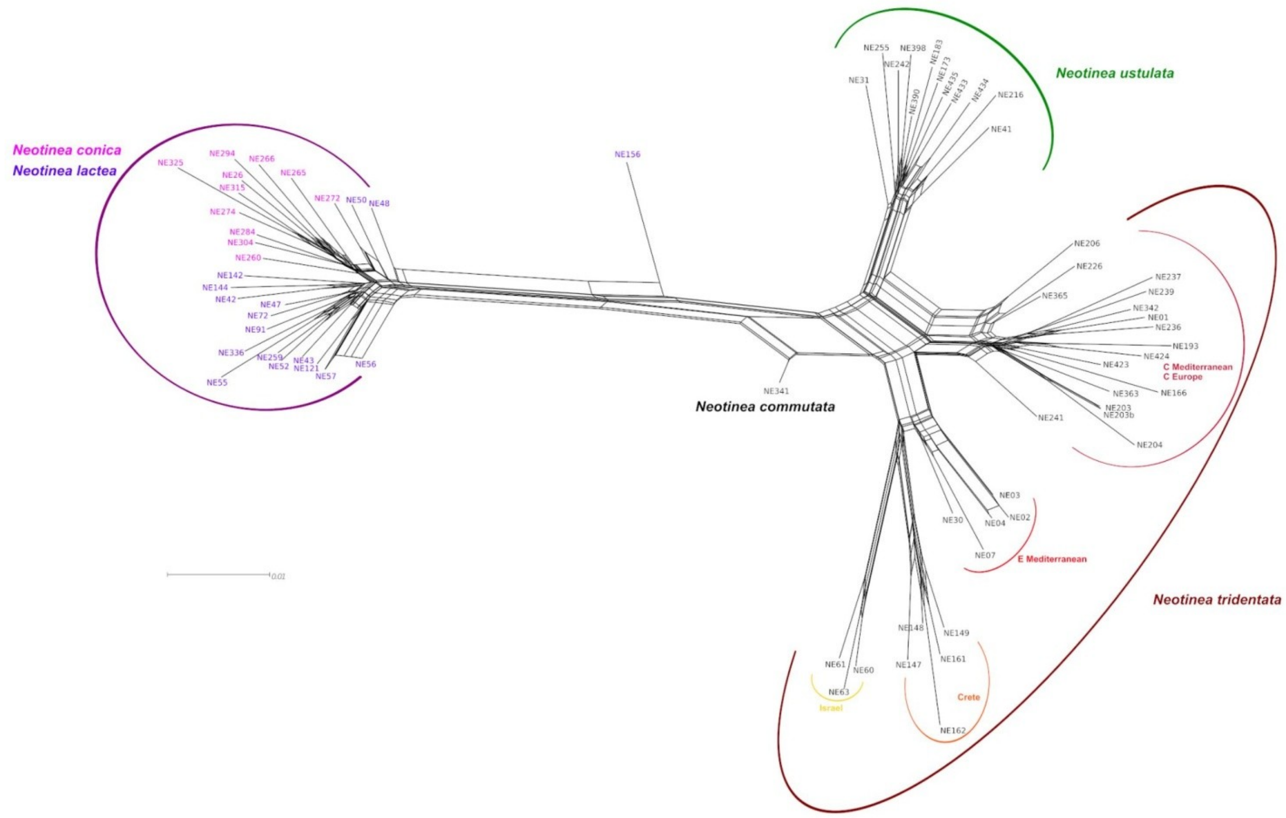

Supplementary Figure 3 | NeighbourNet based on 3,090 random SNPs.

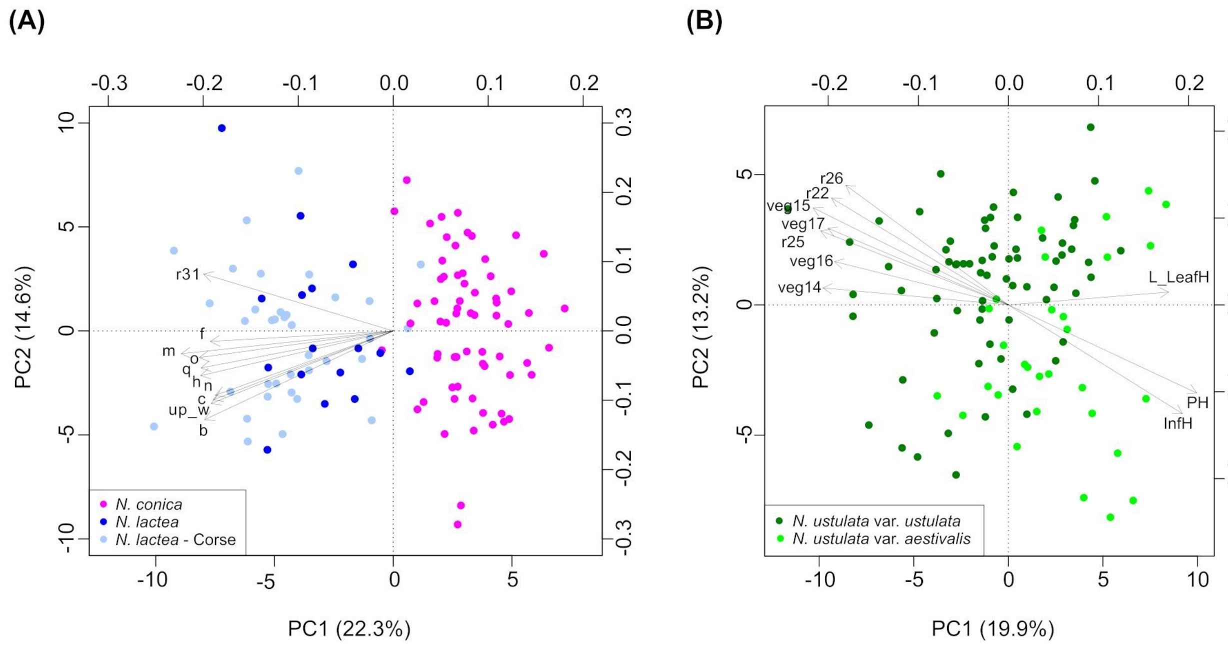

Supplementary Figure 4 | PCA biplot of multivariate morphometrics data on a subset including individuals of the (**A**) *N. lactea* group and (**B**) *N. ustulata* (both varieties).

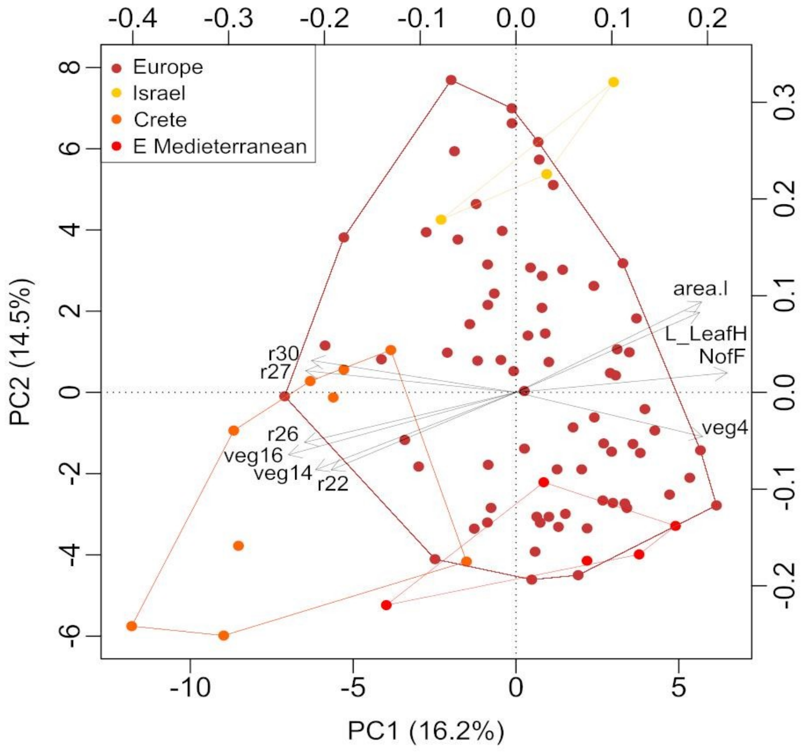

Supplementary Figure 5 | PCA biplot of multivariate morphometrics data on the set of all *N. tridentata* subgroups.
