# Supplementary material for: Integrative Study of Genotypic and Phenotypic Diversity in the Eurasian Orchid Genus Neotinea
Source: Front Plant Sci. 2021 Oct 13;12:734240. doi: 10.3389/fpls.2021.734240 (PMC8570840; doi:10.3389/fpls.2021.734240)
Supplement: Supplementary Table 2 — Overview of morphological characters used for multidimensional morphometry. [file Table_2.docx]

**Supplementary Table 2** – Overview of morphological characters used for multidimensional morphometry. Traits marked with asterisks were excluded due to high correlation (r > 0.95) with other traits.

| **Character code** | **Explanation of the character meaning** | |
| --- | --- | --- |
| PH | above ground height of the plant (cm) | |
| InfH | height of the inflorescence (cm) | |
| InfW | width of the inflorescence (cm) | |
| L_LeafH | length of the longest basal leaf (cm) | |
| L_LeafW | width of the longest basal leaf (cm) | |
| LLWfromEnd | distance of the widest part of the longest basal leaf from its tip (cm) | |
| NofL | number of basal leaves (count) | |
| NofBr | number of stem leaves (count) | |
| SW | width of the stem under inflorescence (cm) | |
| NofF | number of flowers (count) | |
| OvL | length of the ovary (cm) | |
| BrL | length of bracts (cm) | |
| BrW | width of bracts (cm) | |
| veg1 | relative height of the inflorescence (relation to plant height) – InfH / PH | |
| veg2 | the shape of the longest basal leaf (height / width) – L_LeafH / L_LeafW | |
| veg3 | relative position of the widest part of the basal leaf – LLWfromEnd / L_LeafH | |
| veg4 | relative number of flowers (relation to the height of the inflorescence) – NofF / InfH | |
| veg5 | the shape of the inflorescence (height / width) – InfH / InfW | |
| veg6 | relative length of the basal leaf (relation to plant height) – L_LeafH / PH | |
| veg7 | the shape of the bract (length / width) – BrL / BrW | |
| veg8 | relation between height of the inflorescence and number of basal leaves – InfH / NofL | |
| veg9 | relation between height of the inflorescence and number of leaves and stem leaves – InfH / (NofL + NofBr) | |
| veg10 | relation between inflorescence height and the estimate area of all basal leaves – InfH / (area.l * NofL) | |
| veg11 | relation between inflorescence height and estimate area of the longest basal leaf – InfH / area.l | |
| veg12 | relation between shape of the inflorescence and number of basal leves – (InfW / InfH) / NofL | |
| veg13 | relation between shape of the inflorescence and number of all leaves – (InfW / InfH) / (NofL + NofBr) | |
| veg14 | relation between shape of the inflorescence and estimate area of the longest basal leaf – (InfW / InfH) / area.l | |
| veg15 | relation between shape of the inflorescence and plant height – (InfW / InfH) / PH | |
| veg16 | relation between shape of the inflorescence and number of flowers – (InfW / InfH) / NofF | |
| veg17 | relation between shape of the inflorescence and stem width under inflorescence – (InfW / InfH) / SW | |
| veg18 | relative stem width (relation to plant height) – SW / PH | |
| veg19 | relative length of the bracts (relation to the length of the ovary) – BrL / OvL | |
| veg20 | relative width of the bracts (relation to the length of the ovary) – BrW / OvL | |
| area.l | estimate area of the longest leaf – LLWfromEnd*L_LeafW/2 + (L_LeafH-LLWfromEnd)*L_LeafW/2 | |
| ostr | length of spur | all distances are visualised in Supplementary Figure 1 (mm) |
| a* | length of the lip (the shortest maximal length) |  |
| b | width of the lip (the widest part between side lobes) |  |
| c | width of the middle lobe of the lip |  |
| d | length of the side lobe of the lip |  |
| f | width of the lip under side lobes |  |
| g* | length of the middle outer tepal |  |
| h | width of the middle outer tepal |  |
| i* | length of the left outer tepal |  |
| j* | width of the left outer tepal |  |
| k* | length of the right outer tepal |  |
| l* | width of the right outer tepal |  |
| m | width of the narrowest part of the lip |  |
| n | length of the lip from the basis of gynostemium to the narrowing under side lobes |  |
| o | length of gynostemium |  |
| p | length of the lip (the longest maximal length) |  |
| q | height of the side lobe |  |
| up_l | average length of outer tepals – (g + i + k) / 3 | |
| up_w | average width of side outer tepals – (l + j) / 2 | |
| r1 | average shape (width / length) of side outer tepals – (j / i + l / k) / 2 | |
| r2 | average shape (width / length) of middle outer tepal – h / g | |
| r3 | ration between lengths of middle and side outer tepals – g / ((i + k) / 2) | |
| r4 | ration between width of middle and side outer tepals – h / ((j + l) / 2) | |
| r5 | ration between the longest and shortest maximal length of the lip – p / a | |
| r6 | the shape of the flower (overall length / width) – (g + p) / b | |
| r7 | the shape of the lip based on width of the lobes – c / b | |
| r8 | the shape of side lobe of the lip – d / q | |
| r9 | ratio of side lobe and the overall width of the lip – d / b | |
| r10 | ratio between length of the middle lobe and overall length of the lip – (p - n) / p | |
| r11 | ratio between two constrictions of the lip – m / f | |
| r12 | ratio between width of the middle lobe and overall width of the lip – f / c | |
| r13 | the shape of the lip (the longest maximal length / width) – p / b | |
| r14 | the shape of the lip (the shortest maximal length / width) – a / b | |
| r15 | relative length of the gynostemium (relation to the length of the middle outer tepal) – o / g | |
| r16* | relative length of the gynostemium (relation to the length of the lip) – o / p | |
| r17 | relative length of the gynostemium (relation to average length of the lip) – o / ((p + a) / 2) | |
| r18 | relative length of the gynostemium (relation to the narrowest width of the lip) – o / m | |
| r19 | the shape of the lip (ratio between width of the narrowest and widest part of the lip) – m / b | |
| r20 | ratio between the narrowest part of the lip and the width of the middle lobe – m / c | |
| r21 | ratio between the narrowest part of the lip and the width of the middle outer tepal – m / h | |
| r22 | relative length of the outer tepals (relation to plant height) – up_l / PH | |
| r23* | relative length of the lip (relation to the plant height) – p / PH | |
| r24* | relative length of the lip (relation of the average length of the lip and the plant height) – ((p + a)/2) / PH | |
| r25 | relative width of the lip (relation of the width of the narrowest part of the lip and the plant height) – m / PH | |
| r26 | relative width of the lip (relation of the width of the widest part of the lip and the plant height) – b / PH | |
| r27 | relation of the lip width and relative number of flowers – m / (NofF / InfH) = m / veg4 | |
| r28* | relation of the lip width and relative number of flowers – b / (NofF / InfH) = b / veg4 | |
| r29* | relation of the lip length and relative number of flowers – p / (NofF / InfH) = p / veg4 | |
| r30 | relation of the length of the outer tepals and relative number of flowers – ((g + i + k) / 3) / (NofF / InfH) = up_l / veg4 | |
| r31 | relation of the width of the lip (narrowest part) and length of the ovary – m / OvL | |
| r32 | relation of the width of the lip (widest part) and length of the ovary – b / OvL | |
| r33 | relation of the length of the lip and length of the ovary – p / OvL | |
| r34 | relation of the length of the outer tepals and length of the ovary – ((g + i + k) / 3) / OvL = up_l / OvL | |
